# Establishment of clonal myogenic cell lines from severely affected dystrophic muscles - CDK4 maintains the myogenic population

**DOI:** 10.1186/2044-5040-1-12

**Published:** 2011-03-08

**Authors:** Guido Stadler, Jennifer CJ Chen, Kathryn Wagner, Jerome D Robin, Jerry W Shay, Charles P Emerson Jr, Woodring E Wright

**Affiliations:** 1 Department of Cell Biology, UT Southwestern Medical Center at Dallas, Dallas, TX, USA; 2 Boston Biomedical Research Institute, Watertown, MA, USA; 3 Center for Genetic Muscle Disorders, The Kennedy Krieger Institute, Johns Hopkins School of Medicine, Baltimore, MD, USA

## Abstract

**Background:**

A hallmark of muscular dystrophies is the replacement of muscle by connective tissue. Muscle biopsies from patients severely affected with facioscapulohumeral muscular dystrophy (FSHD) may contain few myogenic cells. Because the chromosomal contraction at 4q35 linked to FSHD is thought to cause a defect within myogenic cells, it is important to study this particular cell type, rather than the fibroblasts and adipocytes of the endomysial fibrosis, to understand the mechanism leading to myopathy.

**Results:**

We present a protocol to establish clonal myogenic cell lines from even severely dystrophic muscle that has been replaced mostly by fat, using overexpression of CDK4 and the catalytic component of telomerase (human telomerase reverse transcriptase; hTERT), and a subsequent cloning step. hTERT is necessary to compensate for telomere loss during *in vitro *cultivation, while CDK4 prevents a telomere-independent growth arrest affecting CD56+ myogenic cells, but not their CD56- counterpart, *in vitro*.

**Conclusions:**

These immortal cell lines are valuable tools to reproducibly study the effect of the FSHD mutation within myoblasts isolated from muscles that have been severely affected by the disease, without the confounding influence of variable amounts of contaminating connective-tissue cells.

## Background

Most muscular dystrophies result from a defect within myogenic cells that leads to progressive muscle weakness and wasting, and in severe cases, to the replacement of muscle fibers by connective tissue and/or fat. At advanced stages, skeletal muscle is replaced by fibroblasts and adipocytes. Although these cell types probably promote disease in later stages, it is generally believed that the root cause leading to most muscular dystrophies is a defect originating in myogenic cells. We therefore developed a protocol to enrich, immortalize and isolate rare muscle progenitor cells from severely affected dystrophic muscles to obtain clonal myogenic cell lines. In this report, the protocol is described for cells isolated from skeletal muscle of people with facioscapulohumeral muscular dystrophy (FSHD), but the technique is also applicable to other muscular dystrophies.

FSHD has been linked to deletions of D4Z4 tandem repeats at chromosome 4q [1], but it is still unclear how this deletion causes disease. Primary skeletal-muscle cultures have been used in attempts to model the disease, and several FSHD-specific phenotypes, such as increased vacuolization and sensitivity to oxidative stress [2] have been described. In addition, there have been reports describing incorrect expression of various FSHD candidate genes in muscles and myogenic cells from patients with FSHD, including FSHD region gene (FRG)1 and the double homeobox (DUX)4 gene [3-6]. However, in many instances, these findings have not been consistent between different laboratories, perhaps as a result of variables such as muscle type, disease progression, culture purity, *in vitro *culture conditions or replicative age of the cells. In particular, because of their low replicative potential, it is difficult to use the same purified primary cultures for multiple large-scale experiments in different laboratories to reproduce the findings with the same material. Immortal cell lines provide a solution to this problem, and are a useful and unlimited resource for the research community.

To immortalize FSHD muscle-derived myogenic cells, we used the same strategy as previously described for normal skeletal-muscle cells; that is, ectopic expression of human telomerase reverse transcriptase (hTERT) to overcome replicative senescence, and of CDK4 to block the growth arrest due to cell-culture stress (stress or aberrant signaling-induced senescence (stasis)) [7]. Replicative senescence is an irreversible growth arrest triggered by a DNA damage signal from critically short telomeres [8,9]. Telomere shortening occurs during each cell division, because of the end replication problem [10], end processing [11] and oxidative damage [12], and can be prevented by addition of telomeric repeats by the reverse transcriptase telomerase [13,14]. Overexpression of hTERT, the catalytic subunit of telomerase, has been shown to prevent senescence of a variety of human somatic cells that do not express this gene endogenously [15-17]. However, inadequate *in vitro *culture conditions can cause accumulating stress to certain cell types, which may lead to stasis, a premature growth arrest independent of telomere length. We have shown that overexpression of CDK4 is able to bypass stasis in many cell types, including myoblasts, while maintaining normal phenotypes and cell cycle control [7,18]. We show in this paper that overexpression of CDK4 delays or even prevents overgrowth of myogenic CD56+ cells by their non-myogenic CD56- counterparts, and therefore facilitates the isolation of clonal myogenic cell lines from severely affected dystrophic muscles.

## Results

### Primary cultures from severely affected FSHD muscles primarily consist of non-myogenic cells

**Figure 1:**
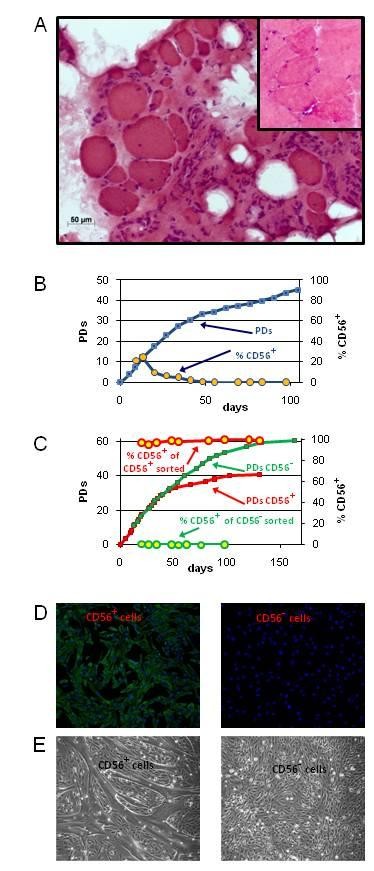
**Replacement of FSHD muscle by non-myogenic cells. (A)** Transverse section of the biceps muscle from subject 01A with facioscapulohumeral muscular dystrophy (FSHD), showing extensive fibrosis and large pockets of adipocytes; muscle fibers displayed variable diameter. By contrast, biceps muscle from his brother (subject 01U, inset) had a relatively uniform array of myogenic fibers with no endomysial fibrosis or fatty infiltration. Hematoxylin and eosin, original magnification 20 ×. **(B) **Percentage of CD56+ cells in primary cell cultures isolated from FSHD biceps muscle shown in (A) during *in vitro *cultivation. PD = population doubling. **(C) **Growth curves of sorted CD56+ (red squares) and CD56- (green squares) populations from the primary 01A biceps cell culture. The percentage of CD56+ cells in sorted, CD56+ and CD56- cultures over time are also displayed (circles outlined in red (CD56+) or green (CD56-)). **(D) **Desmin immunostaining (green) of sorted CD56+ and CD56- cells. Nuclei were counterstained with 4',6-diamidino-2-phenylindole (blue). **(E)** Sorted CD56+ and CD56- cells after 3 days in differentiation medium.

During progression of FSHD, the muscle fibers of certain skeletal muscles are progressively replaced by connective tissue. The biceps is one of the muscles preferentially affected by FSHD (Figure 1A). Despite good strength in the biopsied biceps, muscle fibers from the biceps of a 42-year-old man with FSHD (subject 01A) were found to be in close association with fibrotic tissue and surrounded by large pockets of adipose cells. By contrast, the biceps muscle from his 46-year-old healthy brother (subject 01U, inset) exhibited no endomysial fibrosis, and was virtually free of adipocytes. The percentage of myogenic cells in the primary cell cultures from the biceps muscle of 01A (designated 01Abic) was only 20%, and decreased rapidly during *in vitro *cultivation (Figure 1B). We used CD56 to identify myogenic cells and validated this by several observations. First, CD56+ and CD56- cells were distinct and stable populations (Figure 1C), and skeletal-muscle cells did not switch between a CD56+ and CD56- phenotype. Second, CD56+, but not CD56- cells, expressed the intermediate filament protein, desmin, another marker of skeletal-muscle cells (Figure 1D). Third, purified CD56+ but not CD56- populations fused into multinucleated myotubes when changed to differentiation medium (Figure 1E).

### CDK4 maintains the myogenic population

**Figure 2:**
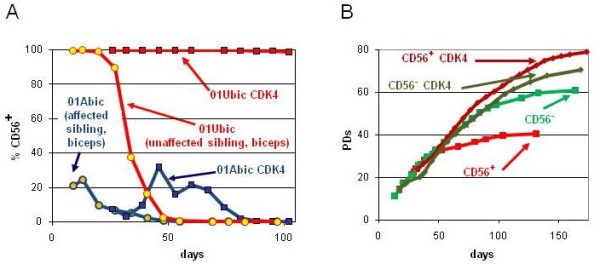
**CDK4 maintains the myogenic population. (A)** Percentage of CD56+ cells in primary cultures from biceps muscle of a subject with facioscapulohumeral muscular dystrophy (FSHD) (01Abic) and his unaffected brother (01Ubic) with and without overexpression of CDK4. **(B) **Growth curves of CD56 sorted 01Abic cells with and without overexpression of CDK4.

To test the hypothesis that stasis of CD56+ cells is responsible for their overgrowth by CD56- cells *in vitro*, we infected primary 01Abic cells with a retrovirus containing CDK4 at the fifth population doubling (PD5), when the fraction of CD56+ cells was about 20%. Whereas the percentage of CD56+ cells quickly dropped towards zero in uninfected cells (Figure 2A, blue line with yellow circles), CDK4 overexpression maintained the CD56+ cells at roughly 20% for an additional 50 days, corresponding to about 25 PDs (Figure 2A, blue line with blue squares). The effect of CDK4 on maintenance of the CD56+ population was also dramatic in the primary cells cultured from the 01U biceps (designated 01Ubic) (Figure 2A, red lines). At early PDs, this cell strain consisted of a seemingly pure myogenic population, which nonetheless was overgrown by initially rare CD56- cells (Figure 2A, red line with yellow circles) at between 30 and 50 days of culture. CDK4 overexpression prevented this overgrowth, and maintained the CD56+ population for the entire culture period (Figure 2A, red line with red squares). These experiments suggest that CDK4 overexpression confers a selective growth advantage on CD56+ cells compared with their CD56- counterparts. This hypothesis was confirmed by infection of CD56 sorted 01Abic cells with CDK4 (Figure 2B). Overexpression of CDK4 led to a doubling of the replicative lifespan of CD56+ cells, from about 40 to 80 PDs. In the absence of CDK4, the growth rate of the CD56+ cells began to slow down as early as PD20, contributing to the overgrowth by CD56- cells long before the CD56+ cells actually had their growth arrested. By contrast, CDK4 had only a minor effect on the lifespan of CD56- cells. Telomeres of CD56- cells were shorter than those of their CD56+ counterparts at growth arrest in the absence of CDK4, consistent with the interpretation that CD56- cells arrested because of replicative aging, whereas CD56+ cells arrested because of stasis before reaching the telomere-based replicative limits. CDK4 overexpression led to further telomere shortening during the increased lifespan of the CD56+ population, reaching lengths similar to those of the senescent CD56- population when they finally became senescent (not shown).

### Myogenic clones uniformly expressing CD56 can be isolated after immortalization of primary cultures consisting mainly of CD56- cells

**Figure 3:**
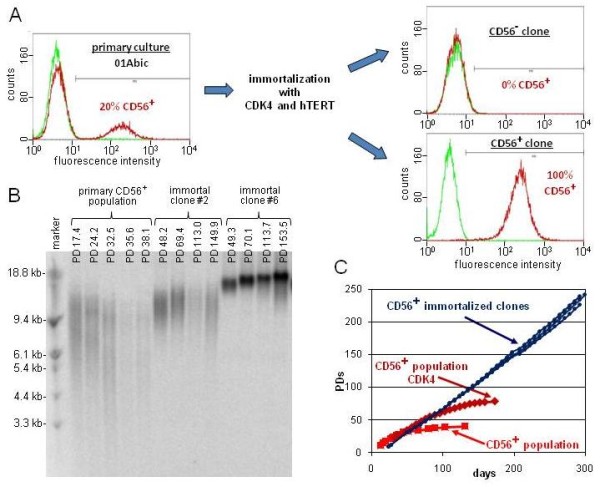
**Isolation of immortal CD56+ clones**. **(A)** FACS histograms of primary 01Abic cells at PD7 (left) and two clones at PD35, one CD56+ and the other CD56-, after immortalization with CDK4 and human telomerase reverse transcriptase (hTERT) (right). Red lines represent cells treated with anti-CD56 antibody; green lines represent controls where the primary antibody was omitted, and hence they correspond to nonspecific background fluorescence. The percentage of CD56+ cells (fluorescence greater than background) is indicated. **(B) **Telomere restriction fragment analysis gel showing telomere length dynamics of the CD56+ population and two immortalized CD56+ clones at different PDs. **(C) **Growth curves of primary 01Abic cells sorted for CD56 (CD56+ population, red squares), cells infected with CDK4 (CD56+ population CDK4, red diamonds) and three CD56+ immortalized clones (blue lines).

Even though pure myogenic populations can be isolated from mixed primary cultures using antibody based methods, such as fluorescence or magnetic-activated cell sorting (FACS or MACS), those cells have a very restricted replicative lifespan, and therefore can be used for only a limited number of experiments. We thus established the following protocol to isolate immortal clonal myogenic cell lines from severely affected dystrophic muscle. Primary cultures (in this case, 01Abic, containing only 20% CD56+ cells) were consecutively infected with retroviruses for overexpression of CDK4 and hTERT, and then uniformly CD56-expressing clones were isolated (Figure 3A). In our hands, differences in viral titers, multiplicity of infection (MOI) and virus type have not had significant effects on rate of immortalization, and we have obtained similar results using low MOI retroviruses or high MOI lentiviruses. For the experiments published here, about 1% of the cells survived selection and therefore had been transduced, corresponding to an MOI of ~0.01. As CDK4 expression maintains the myogenic population, the immortal population had an increased and stable percentage of CD56+ cells after drug selection compared with the parental population at the same time point (14% vs. 2%), and no sorting step before infection was necessary to isolate myogenic clones, which could be carried out easily and with little effort. Healthy-looking and well-separated clones were picked, roughly 90% of which survived the subsequent expansion to the ~2 × 10^6 ^cells necessary for cryopreservation and analysis for myogenicity (CD56 expression and fusion competence). Of 15 randomly analyzed clones, four (27%) were myogenic. The higher percentage of myogenic clones than CD56+ cells in the immortal population (27% vs. 14%) is consistent with the observed growth advantage for CD56+ over CD56- cells at clonal density (data not shown). Growth curves of three immortal myogenic clones are shown in Figure 3C, in comparison with the CD56 sorted primary culture, with and without CDK4. Immortalized myoblasts had a doubling time of about 24 to 30 hours, which we found was only slightly higher than that of primary cultures at early PDs (~20 to 26 hours). All clones underwent >200 PDs without any sign of growth retardation. Whereas telomere restriction fragment analysis showed that telomeres were extended by ectopic hTERT for most myoblast clones (Figure 3B, immortal clone number 6 as a representative example), some clones maintained telomere lengths at levels similar to that of the primary culture (Figure 3B, compare immortal clone number 2 with the primary CD56+ population). The different telomere dynamics may be due to a variety of factors, including hTERT integration site-specific hTERT transcription, or differences in factors affecting telomerase enzyme assembly, recruitment or activity at telomeres.

**Figure 4:**
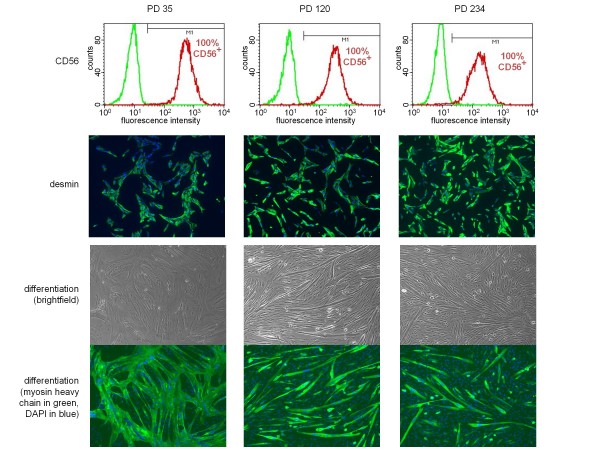
**Characterization of an immortal CD56+ clone during *in vitro *propagation**. Three time points are shown: population doubling (PD) 35, 120 and 234 (see growth curve in Figure 3C). Top row: flow cytometry histograms of CD56 stained cells (red lines) and control cells without the primary antibody (green lines, representing background fluorescence). Uniform expression of CD56 was maintained throughout >200 PDs of *in vitro *cultivation (percentage of CD56+ cells is indicated). Second row: cells stained for expression of the muscle-specific intermediate-filament protein, desmin (green) and counterstained with 4',6-diamidino-2-phenylindole (DAPI) (blue). Third and fourth rows: phase (third row) and fluorescence (fourth row) images of cells in differentiation medium after 3 days (PD 35) or 7 days (PD 120 and PD234) and stained for myosin heavy chain (MF20, green) and DNA (DAPI, blue).

All immortal clones stably expressed CD56 and desmin, and retained their ability to fuse into myosin-expressing, multinucleated myotubes when moved to differentiation medium (Figure 4, 01Abic immortal clone number 6 is shown as a representative example); however, the differentiation kinetics were found to slow down with time in culture. Immortal clones at early PDs (Figure 4, PD35) readily formed very large myotubes after only 3 days in differentiation medium; ~70% of nuclei were present in multinucleated and/or myosin-positive cells, detected by immunostaining with MF20 antibody. By contrast, after >100 PDs (Figure 4, PD120 and PD234), fusion was delayed and resulting myotubes were thinner, with <10% of nuclei present in MF20-positive cells on day 3, and approximately 50% (PD120) and 30% (PD234) appearing in multinucleated/MF20-positive cells at day 7.

## Discussion

Since the description of the first cancer cell line [19], infinitely growing human cell lines have been invaluable tools in biology. In contrast to most cancer cells that are immortal, human somatic cells have a finite lifespan and have to be 'immortalized' to obtain unlimited proliferative capacity. Historically, this was initially achieved by overexpression of viral oncogenes such as simian virus (SV)40 [20], which is always associated with at least some degree of neoplastic transformation. By contrast, hTERT overexpression not only immortalizes a variety of human primary cultures, but has been reported to do this while also maintaining the cellular phenotype [15,21,22]. However, this latter notion has been challenged by the observation of putative non-telomeric activities of hTERT [23-25], with strong evidence for its potential to modulate the Wnt pathway in mice [26]. In most myoblast clones, expression of hTERT for just a few PDs is sufficient to elongate telomeres by several kilobases (Figure 3C, clone number 6). Although the importance of the Wnt pathway in skeletal-muscle differentiation is well documented [27], we have not found any obvious phenotypic change between the hTERT-expressing cells described here and cell models that only transiently overexpress hTERT before Cre recombinase-mediated excision of the hTERT cassette (Stadler and Wright, unpublished data). The slower differentiation kinetics and formation of thinner myotubes seen in high passage hTERT cells (Figure 4) were not due to a direct effect of ectopic hTERT, as this phenotype was not seen with lower passage cells, in which hTERT had already been expressed for >20 PDs. We suggest that this change in differentiation kinetics is instead due to prolonged *in vitro *cultivation and its associated potential for epigenetic drift and constant selection for proliferation. Hence, it is crucial to take the replicative history of a cell into account, even for immortal cell lines, and to perform parallel analyses only on cell lines at roughly similar PD levels. In situations where this is not practical, it may be necessary to confirm that myogenic potential and differentiation kinetics have not changed with additional culturing. These are also important variables to consider when comparing different cell lines and cell clones with one another. Although there may be insertion-specific variation, our findings link changes in the differentiation ability of a cell line with the time spent by that cell line in culture.

The conditions for immortalization of human myoblasts were initially established using primary cultures from the pectoralis major muscle [7]. In addition to cell-culture supplements, such as dexamethasone and hepatocyte growth factor, ectopic CDK4 was necessary to prevent telomere-independent growth arrest (stasis). Fetal lung fibroblasts require the spin-trap compound n-*tert*-butyl hydroxylamine in addition to low oxygen to avoid stasis [28], but this substance was extremely toxic to myoblasts (unpublished observations). This might reflect the consequences of blocking nitric oxide signaling in myoblasts [29].

In fetal fibroblasts and epithelial cells, stasis is often associated with an increase in p16 [18,28], and therefore ectopic expression of CDK4 was chosen to titer out the upregulation of this cell cycle inhibitor. Whereas others have shown that p16 is increased in growth-arresting myoblasts [30], we found that p16 levels are not increased in human myoblasts during stasis [7]. Differential regulation of cell cycle inhibitors *in vitro *may be explained by different culture conditions, or by developmental differences of fetal versus adult myoblasts (used in the former and latter studies, respectively), as has been shown for fibroblasts [28]. We are currently investigating which factors prevent adult myoblasts from being immortalized by hTERT in the absence of CDK4 and are hence responsible for stasis.

In contrast to CD56+ myoblasts, CD56- cells did not exhibit stasis under our culture conditions, which were originally optimized for myoblasts. This is supported by the following observations: First, CDK4 overexpression in CD56- cells did not lead to a significant increase in their proliferative potential. Second, CD56- cells could be immortalized by hTERT alone in conditions of low oxygen (data not shown), suggesting that CD56+ cells are more vulnerable to 'stress' (which triggers stasis) compared with CD56- cells, and therefore CD56- cells may have a growth advantage in 'stressful' situations, such as during pathological regeneration of muscle *in vivo*. Additionally, it seems that at low passage, sorted CD56+ cells have growth rates similar to those of CD56-, whereas the former are rapidly overgrown when unsorted, arguing that CD56- cells might inhibit growth of CD56+ cells. One candidate for this inhibitory activity is myostatin, which has been shown to be expressed by both skeletal-muscle fibroblasts and myoblasts [31], and to inhibit proliferation of myoblasts [32]. Our immortalized CD56+ and CD56- clones from skeletal muscle provide a model system to study the relationship between them and to help elucidate the intercellular crosstalk and regulatory pathways between different cell types.

Although we cannot exclude the possibility that primary cultures from skeletal muscle contain non-myogenic CD56+ cells, such as infiltrating immune cells [33], all CD56+ immortal clones analyzed to date in our laboratory (>20) were desmin-positive and fusion-competent.

## Conclusions

In summary, we describe a simple and efficient protocol to establish immortal myogenic cell lines from dystrophic muscle that consists mainly of non-muscle tissue. Additionally, we provide evidence that stasis of myoblasts and not their CD56- counterpart is responsible for overgrowth of the former by the latter in primary cultures, which can be prevented by ectopic CDK4. To our knowledge, this is the first report of immortal myogenic human cell lines harboring the FSHD mutation, and will be useful in investigating this enigmatic disease.

## Methods

The study was approved by The Johns Hopkins Medicine Institutional Review Board.

### Human subjects and tissue collection

The male proband (subject 01A) first exhibited weakness in the legs as a teenager. At time of enrollment, he was 42 years old, and exhibited mild facial weakness, scapular winging and asymmetric weakness in the limbs, consistent with the FSHD phenotype. Molecular diagnosis of FSHD was confirmed by the University of Iowa Diagnostic Laboratories, which identified a *Hin*dIII 4qA allele of 26 kb in length. An open muscle biopsy was taken from the biceps muscle (01Abic), which had its strength rated as 4+ out of 5 on the modified Medical Research Council scale. Tissue was also obtained from the biceps muscle (01Ubic) of the proband's 46-year-old brother (01U), who had full muscle strength and no disease allele found on gene testing. Approximately 300 mg of tissue was reserved for histological and biochemical assays, and ~500 to 700 mg tissue was used for primary cell isolation.

### Primary cell isolation and cell culture

The biceps muscle tissue was minced into fragments of <5 mm^2^, and stored at 4°C in a Ham's F10 medium containing 20% fetal bovine serum (FBS; Hyclone, Thermo Scientific, Logan, UT, USA), 2% chick embryo extract and 2.5 ng/ml basic fibroblast growth factor (Biopioneer, San Diego, CA, USA), supplemented with 1% antibiotic/antimycotic (Cellgro, Manassas, VA, USA) until processed for cell isolation (within approximately 24 hours of biopsy) (viable cells have been successfully isolated from tissues stored in this manner up to 6 days after biopsy). Tissue was cleaned and minced in Hanks' balanced salt solution (HBSS, catalogue number 14175; Gibco, Grand Island, NY, USA) and dissociated in 5 ml of enzyme solution (1 mg/ml collagenase IV, 2.4 U/ml dispase (both Worthington, Lakewood, NJ, USA) and 2.5 mmol/l CaCl_2 _in HBSS] for 45 minutes at 37°C, triturating every 15 minutes with a 5 ml serological pipette (moving it up and down ~10 to 15 times), and filtered through 100 μm and 40 μm cell sieves (BD Biosciences, Bedford, MA, USA). Cells were pelleted by spinning in a centrifuge at 1000 *g *for 5 minutes, and resuspended in 2 ml growth medium (Ham's F-10 supplemented with 20% FBS (Hyclone), 0.5% chick embryo extract, 1.2 mmol/l CaCl_2 _and 1% antibiotic/antimycotic (Cellgro), prepared as fresh stock at least every 7 days) and counted with a hemocytometer. The 01Abic cells were seeded in 2 ml growth medium on a 35 mm gelatin-coated dish, and the 01Ubic cells were seeded on a 60 mm dish in 5 ml growth medium. Cells were cultured undisturbed for 48 hours, after which they were given fresh growth medium daily for 2 to 4 days. When approximately 50 to 70% confluent, cells were treated with trypsin (TrypLE Express/Gibco) for 5 minutes at 37°C, neutralized with an equal volume of growth medium, and counted with a hemocytometer. For expansion, cells were seeded at ~2000 to 3000 cells/cm^2 ^in growth medium. For freezing, an equal volume of ice-cold 2 × freezing medium (20% dimethysulfoxide, 50% FBS, 30% growth medium) was added to cells and mixed well, then cells were incubated in the liquid nitrogen vapor phase for at least 1 hour before storage at -140°C.

To standardize conditions for growth of primary and immortalized cells, primary myoblasts were subsequently cultured in a 4:1 mixture of Dulbecco modified Eagle medium (DMEM) and Medium 199 (Hyclone), supplemented with 15% FBS (Hyclone), 0.02 M HEPES buffer, 1.4 mg/l vitamin B12 (both Sigma-Aldrich, St. Louis, MO, USA), 0.03 mg/l ZnSO_4 _(Fisher Scientific, Fair Lawn, NJ, USA), 0.055 mg/l dexamethasone (Sigma-Aldrich), 2.5 μg/l hepatocyte growth factor (Chemicon International, Temecula, CA, USA) and 10 μg/l basic fibroblast growth factor (BioPioneer), on dishes coated with 0.1% pigskin gelatin (Sigma-Aldrich) in a 2-5% oxygen environment [34]. Hepatocyte and fibroblast growth factors were added to a volume of medium that was used within 1 week. Cells were routinely grown in 100 mm tissue culture dishes (BD Falcon, Franklin Lakes, NJ, USA) covered by 10 ml medium, with a weekly medium change if cells were not passaged before that (usually one passage every 5 to 7 days). Cells were passaged at 50 to 90% confluency using 0.05% trypsin/EDTA (Gibco), and cell numbers determined (Z1 Coulter Particle Counter; Beckman Coulter, Miami, FL, USA). PD was calculated using the formula: PD = ln[(final number of cells)/(initial number of cells)]/In(2).

For differentiation, 1.2 × 10^5 ^myoblasts were seeded in six-well dishes, then 24 hours later, cells were washed twice with phosphate-buffered saline (PBS) and fed with differentiation medium (DMEM and Medium 199 in a ratio of 4:1, supplemented either with 2% horse serum (Gibco) or with 0.02 mol/l HEPES plus 10 mg/l insulin and 100 mg/l apo-transferrin (both Sigma-Aldrich)). Results obtained were similar with both differentiation media, although a delay of ~1 day was observed with horse serum relative to insulin/transferrin.

### Retroviral infection

The construction of the vectors used for immortalization (CDK4-pBabe-neo and hTERT-pBabe-Hygro), has been described previously [7,18]. In brief, mouse CDK4 (kindly provided by Charles J. Sherr) and hTERT cDNAs were inserted into pBabe vectors [35] containing neomycin- and hygromycin-resistance genes, respectively. Additionally, loxP sites were placed internal to the long terminal repeats of the hTERT expression vector, to allow for excision of the entire expression cassette by Cre recombinase. These vectors were transfected into the Phoenix ecotropic packaging cell line using the calcium phosphate technique [36], and the virus-containing supernatant was used to infect the amphotropic packaging cell line PA317 [37] to obtain stable virus-producing cell lines after selection with 0.5 mg/ml G418 or hygromycin (EMD Biosciences, San Diego, CA, USA). All infections were performed in the presence of 2 μg/ml polybrene (Sigma-Aldrich).

For infection of myoblasts, 5 × 10^4 ^cells were seeded in six-well dishes 24 hours before infection. Selection was started 48 hours after infection using 300 mg/l hygromycin and 400 mg/l G418 for 1 and 2 weeks, respectively. Depending on growth rates and infection efficiency, cells were passaged during the selection period before becoming confluent.

### Isolation of myogenic immortalized clones

Immortalized populations were seeded at low densities, (50, 100, 200 or 400 cells per 100 mm dish). About 10 days after seeding, clones were isolated using cloning rings and seeded into 48-well dishes, followed by six well and 100 mm dishes. The exact time point for clone isolation was chosen to maximize the cell number and minimize cell density of individual clones, to facilitate growth after passage but avoid fusion of myoblasts, respectively. We estimate that clones contained 100 to 1000 cells at the time of isolation. All clones were analyzed for CD56 expression by flow cytometry (see below) and fusion potential in differentiation medium. The percentage of cell nuclei in multinucleated and/or MF20-positive cells was determined for 10 random fields after 3 or 7 days in differentiation medium.

### Terminal restriction fragment assay

Terminal restriction fragment assay was performed as previously described [38].

### Flow cytometry

Cells were detached with trypsin, counted, and sedimented by centrifugation, then 1.5 × 10^5 ^cells were resuspended in 0.2 ml DMEM with 10% calf serum (Cosmic Calf Serum; Hyclone) containing 5% supernatant from the hybridoma line 5.1H11 [39], which recognizes CD56 (kindly provided by Helen Blau). As a control, primary antibody was omitted. Cells were incubated for 30 min at room temperature, washed twice with 10% calf serum (Cosmic Calf Serum; Hyclone) in PBS, and incubated with secondary Alexa488-conjugated goat anti-mouse antibody (Invitrogen, Eugene, OR, USA) for 30 min at room temperature. After washing with PBS, cells were resuspended in 0.2 ml PBS and analyzed on a flow cytometer (FACSCalibur; Becton Dickinson). A second flow cytometer (FACSAria; Becton Dickinson) was used for sorting, with 5 × 10^6 ^cells stained in upscaled volumes.

### Immunofluorescence

Cells on gelatin-coated glass chamber slides (Thermo Scientific) were fixed with cold (-20°C) ethanol for 5 minutes, air-dried, and rehydrated with PBS. After blocking with 10% calf serum (Cosmic Calf Serum; Hyclone) in PBS, slides were incubated with primary antibody (1:100 monoclonal mouse anti-desmin clone D33 (Thermo Scientific, Rochester, NY, USA) or MF20 supernatant (myosin heavy chain, 1:30; Developmental Studies Hybridoma Bank, Iowa City, IA, USA) for 1 hour at room temperature. Slides were washed three times with PBS for 5 min, each and incubated with secondary Alexa488-conjugated goat anti-mouse antibody (Invitrogen) for 1 hour at room temperature. After three washes with PBS, slides were covered with mounting medium containing 4',6-diamidino-2-phenylindole (Vectashield; Vector Laboratories, Burlingame, CA, USA) and imaged with a fluorescence microscope (Axiovert 200 M; Zeiss).

### Histology

Tissues were frozen in liquid nitrogen-cooled isopentane, and cut on a cryostat. Frozen sections (0.8 μm) were collected on microscope slides (Fisherbrand Superfrost Plus; Fisher Scientific), air-dried, and fixed with 4% formaldehyde in PBS for 15 minutes at room temperature. Slides were washed three times with PBS and stained with hematoxylin and eosin. Sections were imaged under a microscope (AxioImager M2, Zeiss) and images captured with a camera (Digital microscopy camera Axio Cam, ICC3, Zeiss).

## Competing interests

The authors declare that they have no competing interests.

## Authors' contributions

KW, CPE and WEW designed this study. GS, JC, KW and JR performed the experiments and analyzed the results. GS, JC, KW, JWS and WEW wrote the manuscript. All authors read and approved the final manuscript.
